# Donor-Derived Vγ9Vδ2 T Cells for Acute Myeloid Leukemia: A Promising “Off-the-Shelf” Immunotherapy Approach

**DOI:** 10.3390/cancers17193166

**Published:** 2025-09-29

**Authors:** Amanda Eckstrom, Anudishi Tyagi, Maryam Siddiqui, Jenny Borgman, Jieming Zeng, Adishwar Rao, Abhishek Maiti, Venkata Lokesh Battula

**Affiliations:** 1 Department of Leukemia, The University of Texas MD Anderson Cancer Center, Houston, TX 77030, USA; ageckstrom@mdanderson.org (A.E.); anudishi.tyagi@vcuhealth.org (A.T.); msiddiqui3@mdanderson.org (M.S.); jmborgman@mdanderson.org (J.B.); adishwar.rao@guthrie.org (A.R.); amaiti@mdanderson.org (A.M.); 2 Department of Internal Medicine, Massey Comprehensive Cancer Center, Virginia Commonwealth University, Richmond, VA 23298, USA; 3 CytoMed Therapeutics Limited, Singapore 149544, Singapore; jiemingzeng@cytomed.sg; 4 Department of Breast Medical Oncology, The University of Texas MD Anderson Cancer Center, Houston, TX 77030, USA

**Keywords:** Vγ9Vδ2 T cells, acute myeloid leukemia, adoptive cell therapy, venetoclax

## Abstract

Background: Venetoclax-based combination therapies have provided treatment options for patients with acute myeloid leukemia (AML) who are unfit for intensive chemotherapy. However, venetoclax resistance is common, and for such patients, the prognosis is dismal, and treatment approaches with different mechanisms of action are urgently needed. γδ T cells are a promising candidate owing to their good safety profile and cytotoxic effects in various types of cancers but are mostly unstudied in AML. Methods: Here we used flow cytometry to profile the subtype and memory phenotype of peripheral blood γδ T cells in AML patients and investigate the feasibility of using donor-derived Vγ9Vδ2 T cells to treat AML as both a single agent and in combination with venetoclax. Additionally, we used bioluminescence imaging to examine the effect of donor-derived Vγ9Vδ2 T cells on AML xenograft models alone and in combination with venetoclax. Results: We observed that Vδ2 T cells were less abundant and the TEMRA (terminally differentiated effector memory) phenotype was more prevalent as compared with that of healthy donors, suggesting that replenishing patients with Vδ2 T cells may be an effective treatment option. We found that donor-derived Vγ9Vδ2 T cells that Vγ9Vδ2 T cells efficiently induced apoptosis in AML cells from eight cell lines and three primary cultures in an effector-to-target cell ratio-dependent manner. Moreover, Vγ9Vδ2 T cells showed potent cytotoxicity against the venetoclax-resistant OCI-AML3 cell line and remained potent in the presence of venetoclax. Treatment with Vγ9Vδ2 T cells significantly extended survival in two AML xenograft models established with the aggressive Molm-13 and the venetoclax-resistant OCI-AML3 cell lines. An additive effect of venetoclax and Vγ9Vδ2 T cells was observed in the latter model. Conclusions: Overall, these findings suggest Vγ9Vδ2 T cells as a promising “off-the-shelf” immunotherapy approach for AML patients, especially for patients with venetoclax-resistant disease.

## Simple Summary

Donor-derived Vγ9Vδ2 T cells demonstrate potent anti-leukemic activity both in vitro and in vivo, offering a promising immunotherapeutic approach for acute myeloid leukemia (AML). These γδ T cells efficiently induce apoptosis in AML cells in vitro, highlighting their direct cytotoxic potential. In an aggressive AML xenograft mouse model, treatment with donor-derived Vγ9Vδ2 T cells significantly prolonged survival, indicating their therapeutic relevance in a clinically challenging setting. Importantly, these cells remain effective against venetoclax-resistant AML; in vivo studies further show that Vγ9Vδ2 T cells extend survival in mouse survival with or without venetoclax. These findings suggest the therapeutic potential of harnessing donor-derived Vγ9Vδ2 T cells as a novel strategy to overcome drug resistance and improve outcomes in AML, including in patients with limited responses to existing therapies.

## 1. Introduction

Acute myeloid leukemia (AML) is the second most common leukemia in adults, representing about 80% of acute leukemia cases with an incidence rate of 4.3 per 100,000 people each year [1,1]. Cure rates for AML are 40% for patients under the age of 60 years but only 15% for patients above the age of 60, an especially bleak outlook considering the median age of AML onset is 68 [2,3]. The 7 + 3 intensive chemotherapy regimen, named for its intravenous infusion of cytarabine for 7 days and daunorubicin for 3 days, is the standard of care for AML [4]. However, over 50% of newly diagnosed patients are ineligible for such intensive chemotherapy due to comorbidities or older age, and the treatment options for these patients are limited [5]. The recent advent of venetoclax, a BCL2 inhibitor, has transformed the treatment landscape for these patients, and its combinations with hypomethylating agents (VEN + HMA) have become standard treatments [6,7]. Unfortunately, even with the improvement to the treatment landscape that venetoclax offers, the median overall survival for patients with venetoclax-sensitive disease remains around 15 months, and in patients with venetoclax-resistant or relapsed AML, the median overall survival is less than 3 months after VEN + HMA treatment [8,9], which stresses the urgent need for a novel therapeutic approach.

Autologous T cell therapies, especially CAR-T therapy, have been successful in treating hematologic malignancies, but several challenges remain, including antigen escape and high rates of treatment-related toxicities [10,11]. Moreover, these autologous therapies are also expensive, costing an estimated USD 400,000 to USD 1 million for each patient, and require several weeks to produce; as many as 30% of patients are unable to receive the treatment due to the time required to produce it [12]. In contrast, allogeneic cell therapy based on γδ T cells is less expensive and offers the potential for a quicker “off-the-shelf” treatment option [13]. γδ T cells differ from the more prevalent αβ T cell subset in their tissue tropism and target recognition [14,15]. γδ T cells are a key immune defense against virally infected and malignant cells, recognizing stress-induced molecules in a major histocompatibility complex (MHC)-independent manner [14]. In a meta-analysis of gene expression in about 18,000 human tumors representing 39 different cancers, infiltration of γδ T cells was identified as the best prognosticator for patient survival [16]. In AML patients after stem cell transplantation, higher γδ T cell reconstitution has been associated with improved graft-versus-leukemia effect, leukemia-free survival, overall survival, and reduced risk of relapse [17,18,19,20].

γδ T cells are categorized based on the structure of the variable regions of the T cell receptor (TCR) into two main subsets, Vδ1 T cells and Vδ2 T cells [21]. Vδ1 T cells have been observed in the peripheral blood of patients with chronic lymphocytic leukemia (CLL) and previous studies have found that ex vivo-expanded, peripheral blood-derived Vδ1 T cells are cytotoxic against B-cell CLL cells [22,23]. However, the role of Vδ1 T cells in cancer is controversial, as they have also been found to have tumor-promoting roles and have been implicated in inflammation-induced cancer progression [24]. Imbalance in the Vδ1-to-Vδ2 T cell ratio, specifically a greater proportion of Vδ1 cells, has been associated with cancer development in melanoma and rectal cancer [25,26,27]. In comparison, Vδ2 T cells are the most common γδ T cell subtype in the peripheral blood, and most Vδ2 T cells express TCRs with the Vδ2 chain paired with the Vγ9 chain (Vγ9Vδ2 TCR); thus, these cells are also known as Vγ9Vδ2 T cells [28]. In addition to their ability to recognize stress markers, Vγ9Vδ2 T cells can also recognize accumulated phosphoantigens (pAgs) in cells with dysregulated mevalonate metabolism, which is common in malignant cells [28,29,30]. Furthermore, previous studies have found that lower Vδ2 T cell concentrations following stem cell transplant in AML patients are associated with higher mortality rates after 2 and 5 years [31], and a recent phase I clinical trial, TCB-202-001, observed improved outcomes in the patients treated with allogeneic ex vivo expanded Vγ9Vδ2 T cells [32].

Based on CD27 and CD45RA expression, γδ T cells can be further divided into various memory phenotypes: naïve, central memory (CM), effector memory (EM) and terminally differentiated effector memory (TEMRA) [33,34,35]. The memory phenotype profile may change in disease conditions, e.g., in some chronic lymphocytic leukemia patients, γδ T cells are predominantly the TEMRA phenotype and less proliferative [36]. Therapies harnessing endogenous γδ T cells in AML patients have recently shown benefit in a frontline therapy setting [37], but the low response in some patients may be attributed to γδ T cell dysfunction and exhaustion. This highlights the opportunity of harnessing allogeneic Vγ9Vδ2 T cells as a treatment for AML. In this study, we sought to profile the peripheral blood γδ T cell population of AML patients unfit for intensive chemotherapy and investigate the potential of adoptive transfer of donor-derived Vγ9Vδ2 T cells as immunotherapy for AML.

## 2. Materials and Methods

### 2.1. Cell Culture

AML cell lines U937 (CRL-1593.2), THP1 (TIB-202), Kasumi-1 (CRL-2724), and MV4-11 (CRL-9591) were purchased from ATCC, and the AML cell lines OCI-AML2 (ACC 99), OCI-AML3 (ACC582), Molm-13 (ACC 554), and Molm-14 (ACC 777) were obtained from DSMZ. All AML cell lines were cultured in RPMI1640 media (Corning (Corning, NY, USA), 15-040-CV) supplemented with 1% L-glutamine (Corning, 25-005-CI), 1% penicillin/streptomycin (Sigma-Aldrich (St. Louis, MI, USA), P4333), and 10% fetal bovine serum (Gibco (Waltham, MA, USA), 26140-079). The culture medium was changed twice weekly. *Mycoplasma* contamination was tested every 4 to 6 months.

### 2.2. Peripheral Blood PBMCs and Primary AML Cells

For peripheral blood γδ T cell profiling, frozen PBMC samples from AML patients were obtained from the MD Anderson Cancer Center Leukemia Sample Bank, and these samples were collected from December 2014 to October 2024 from de novo AML patients enrolled on a hypomethylating agent and venetoclax combination (HMA–Ven) protocol. As a control, fresh peripheral blood samples from healthy 30- to 70-year-old donors were obtained from Gulf Coast Regional Blood Center (Houston, TX, USA) from February 2023 to August 2024. PBMCs were isolated using density gradient centrifugation and frozen in a solution of 10% dimethyl sulfoxide and 90% fetal bovine serum in a liquid nitrogen tank until use.

To generate primary AML cells, fresh peripheral blood samples were collected from 3 patients between December 2024 and January 2025. Primary AML cells were isolated from these samples using density grade centrifugation and were cultured in StemSpan leukemic cell culture medium consisting of 90% StemSpan SFEM II (STEMCELL Technologies (Vancouver, BC, Canada), 09605), 10% StemSpan CD34+ Expansion Supplement Mix (10×) (STEMCELL Technologies, 02691), and 1 µM UM729 (STEMCELL Technologies, 72332) for up to 48 h before use for the flow cytometric apoptosis assay.

All patient sample collections were conducted according to a protocol approved by the Institutional Review Board at The University of Texas MD Anderson Cancer Center (Protocol PA18-0129). All study participants provided written informed consent. Patient characteristics were extracted from MD Anderson Cancer Center’s electronic health record system (EPIC, Epic Systems Corporation (Verona, WI, USA)) and are shown in Supplementary Table S1.

### 2.3. GMP-Grade Donor-Derived Vγ9Vδ2 T Cell Generation

Frozen donor-derived Vγ9Vδ2 T cells were provided by CytoMed Therapeutics Limited (Singapore). The cells were expanded from healthy donor PBMCs and cryopreserved according to the established SOPs in CytoMed’s GMP facility as described by Du et al. [38]. Written informed consent has been obtained from all PBMC donors. Upon request, these GMP-grade frozen Vγ9Vδ2 T cells were shipped to our lab in dry ice and stored in a liquid nitrogen tank until use.

### 2.4. Flow Cytometric Profiling of γδ T Cells

**Figure 1:**
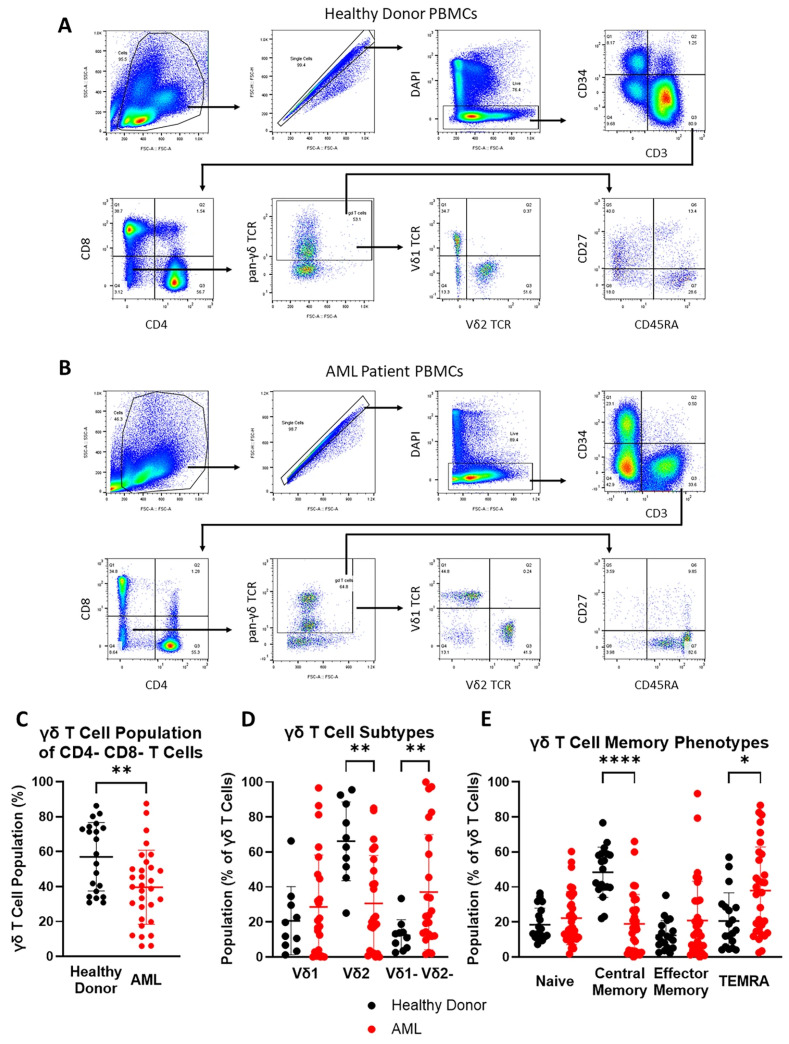
Subtype and memory phenotype profile of peripheral blood γδ T cell population of AML patients. Panels (**A**,**B**): Representative plots showing the gating strategy to identify various T cell populations in peripheral blood of healthy donors and AML patients (UTH5225). Panel (**C**): Scatter plot showing the percentage of γδ T cells in CD4-CD8- T cells among PBMCs from a healthy donor (*n* = 21) and an AML patient (*n* = 32). Panel (**D**): Scatter plot showing the prevalence of Vδ1 and Vδ2 subtypes in γδ T cells among PBMCs from healthy donors (*n* = 10) and AML patients (*n* = 24). Panel (**E**): Scatter plot showing the memory phenotype profile of peripheral blood γδ T cells in healthy donors (*n* = 21) and AML patients (*n* = 32). All fluorochrome markers in the flow cytometry dot plots (**A**,**B**) are represented in hyperlogarithmic scale, and all scatter plots (**C**–**E**) show mean and SD. * *p* ≤ 0.05, ** *p* ≤ 0.01, **** *p* ≤ 0.0001.

To examine the γδ T cell populations in AML patients, we developed a multi-color flow cytometry panel using 4′,6-diamidino-2-phenylindole (DAPI), CD34-BV650 (Biolegend (San Diego, CA, USA), 343624), CD3-BV570 (Biolegend, 300436), CD4-Kiravia Blue 520 (Biolegend, 344660), CD8-APC (Biolegend, 344722), pan-γδ TCR-PE (Biolegend, 331210), CD27-PE/Cy7 (Biolegend, 356412), CD45RA-APC Fire 750 (Biolegend, 304152), TCR Vδ1-PerCP Vio 700 ReAfinity (Miltenyi Biotec (Bergisch Gladbach, Germany), 130-120-441), and TCR Vδ2-Alexa Fluor 700 (Biolegend, 331416). The viabilities of the thawed AML patient-derived PBMCs are shown in Supplementary Table S2. These were determined using 4′,6-diamidino-2-phenylindole (DAPI) staining of the single-cell population within the lymphocyte gate, as shown in our gating strategy in Figure 1A,B. All antibodies for surface staining were added simultaneously at the same concentration using the same dilution of 3 µL of antibody per 200 µL total sample volume as recommended by the manufacturers. CD27 and CD45RA positivity were used to determine memory phenotype. Frozen PBMCs were thawed and washed with phosphate-buffered saline (PBS) (Corning Inc. (Corning, NY, USA), 21-040-CMR) before staining per the manufacturers’ instructions, and flow cytometric analysis was performed on the Miltenyi Biotec MacsQuant16.

### 2.5. Flow Cytometric Apoptosis Assays

AML cell lines (OCI-AML2, OCI-AML3, U937, THP1, Kasumi-1, MV4-11, Molm-13, and Molm-14) and primary AML cells (from 3 AML patients) were used as target cells. The cells were seeded in a Falcon flat-bottom 96-well plates (Corning Inc. (Corning, NY, USA), 353072) at a density of 4 × 10^4^ cells in 100 µL per well (4 × 10^5^ cells/mL). AML cells and freshly thawed donor-derived Vγ9Vδ2 T cells were counted using the Beckman Coulter Vi-CELL BLU cell viability analyzer (Beckman Coulter, Brea, CA, USA), which operates based on the Trypan Blue Dye Exclusion method. The percentage of viable cells was recorded, and experiments were only performed with AML cells with at least 85% viability and Vγ9Vδ2 T cells with at least 80% viability. Media (control) or Vγ9Vδ2 T cells at effector-to-target cell (E:T) ratios of 2:1, 5:1, and 10:1 were added to the plate. The AML cells and Vγ9Vδ2 T cells were co-cultured for 18–20 h. Each sample was then transferred to a round-bottom 96-well plate for staining with CD33-PE (Biolegend 303404), CD3-APC (Biolegend 300439), Annexin-V-FITC (Biolegend 640945), and DAPI. The staining and washing were performed in Annexin-V binding buffer (Biolegend, 422201). Flow cytometric analysis was performed using a Miltenyi Biotec MACSQuant16 flow cytometer (Miltenyi Biotec, Bergisch Gladbach, Germany).

### 2.6. Vγ9Vδ2 T Cell Therapy in Molm-13 GFP Luc AML Xenograft Model

To examine the potential of Vγ9Vδ2 T cell therapy in vivo, we used a green fluorescent protein- and luciferase-expressing Molm-13 cell line (Molm-13 GFP *Luc*) to establish an AML xenograft model in male NOD/scid gamma (NSG) mice (Jackson Laboratory (Bar Harbor, ME, USA)). All the mice were housed in the Animal Core Facility at the MD Anderson Cancer Center. All mouse experiments were performed in accordance with MD Anderson Institutional Animal Care and Use Committee (IACUC) guidelines and were approved by the committee. The Molm-13 GFP *Luc* cells were generated via a retroviral vector encoding the enhanced green fluorescent protein firefly luciferase (eGFP-FFluc) gene [39]. 0.5 × 10^6^ Molm-13 GFP *Luc* cells were injected into each mouse via tail vein, and the leukemia engraftment was monitored through bioluminescence imaging on a Spectral Instruments Imaging Ami imaging system. Once engraftment reached a minimum of 1 × 10^6^ lumens, the mice were randomized into 4 groups with 5 mice per group and treated with 1 × 10^6^, 5 × 10^6^, or 1 × 10^7^ Vγ9Vδ2 T cells or vehicle (PBS) through tail vein injection once a week for 4 weeks. Mice were euthanized when they became moribund.

### 2.7. Vγ9Vδ2 T Cells and Venetoclax Combination Therapy in OCI-AML3 GFP Luc AML Xenograft Model

To investigate the combinatorial effects of Vγ9Vδ2 T cell therapy with venetoclax in vivo, a GFP- and luciferase-expressing OCI-AML3 cell line (OCI-AML3 GFP *Luc*) was used to establish an AML xenograft model in NSG mice. 1.5 × 10^6^ OCI-AML3 GFP *Luc* cells were injected into each mouse via tail vein, and the leukemia engraftment was monitored through bioluminescence imaging on a Spectral Instruments Imaging Ami. Once the minimum engraftment radiance of 1 × 10^6^ lumens was reached, the mice were randomized into 4 treatment groups with 8 mice per group. Mice were then treated for 6 weeks with 100 mg/kg venetoclax (Adooq Bioscience (Irvine, CA, USA), A12500) or vehicle (PBS) through oral gavage twice weekly and with 5 × 10^6^ Vγ9Vδ2 T cells or vehicle (PBS) through tail vein injection once weekly. Mice were euthanized when they became moribund.

### 2.8. Statistical Analysis

Flow cytometry plots were generated using Miltenyi Biotec MACSQuantify (https://www.miltenyibiotec.com/, last accessed 1 August 2024) and FlowJo v10.10.0 software; GraphPad Prism 10 was used to generate all other figures and for statistical analysis. *p*-values of 0.05 or lower were considered statistically significant. All flow cytometric data was acquired and analyzed on a hyperlogarithmic scale and is reported as mean ± standard deviation (SD). One-way Welch ANOVA with multiple comparisons was used to compare the absolute live cell count and percentage of apoptotic leukemic cells across treatment groups for all flow cytometric apoptosis assays. Two-way ANOVA was used to determine the interaction effect between Vγ9Vδ2 T cells and venetoclax in the flow cytometric apoptosis assay. For the in vivo experiments, leukemia engraftment was measured by the radiance calculated through ROI analysis performed using Spectral Instruments Aura64 software (https://spectralinvivo.com/software/, last accessed 1 January 2025) and compared between treatment groups using two-way ANOVA with multiple comparisons tests. Survival was compared between treatment groups using log-rank tests.

## 3. Results

### 3.1. Vδ2 T Cells Are Less Abundant and TEMRA Phenotype Is More Prevalent in Peripheral Blood γδ T Cell Population of AML Patients

To study the subtype and memory phenotype profile of γδ T cells in peripheral blood from AML patients, PBMCs from healthy donors and selected AML patients on HMA–Ven protocols were collected and analyzed using flow cytometry. Figure 1A,B show the gating strategy used to identify various T cell populations in PBMCs, and Table 1 summarizes the mean percentage of each population. The distribution of the γδ T cell profiling data was parametric (D’Agostino and Pearson, 0.7050). Notably, the mean percentage of CD8+ T cells was significantly lower in AML patients than that in healthy donors (27.97% ± 13.74% vs. 41.32% ± 9.29%; *p* = 0.0004), while the mean percentage of CD4-CD8- T cells was significantly higher (14.84% ± 19.37% vs. 5.00% ± 2.90%; *p* = 0.031) (Table 1 and Supplementary Figure S1). However, within the CD4-CD8- T cells, the percentage of γδ T cell population was significantly smaller in AML patients than in healthy donors (39.62% ± 21.14% vs. 57.03% ± 19.6%; *p* = 0.0039) (Figure 1C).

Subtype analysis of the γδ T cell population (Figure 1D) showed that the Vδ1-Vδ2-γδ T cell population is more dominant in AML patients compared with that in healthy donors (37.09% ± 32.96% vs. 12.37% ± 9.042%; *p* = 0.006), while the Vδ2 T cell population is less dominant (30.59% ± 27.46% vs. 66.21% ± 22.54%; *p* = 0.0023). Memory phenotype profiling showed that the CM γδ T cell population is significantly lower in AML patients compared to that in healthy donors (18.95% ± 16.78% vs. 48.45% ± 14.36%; *p* < 0.0001), while the TEMRA population was more prevalent (37.94% ± 24.94% vs. 20.63% ± 16.10%; *p* = 0.0163) (Figure 1E). The lesser abundance of Vδ2 T cells as well as the prevalence of the TEMRA phenotype in the peripheral blood γδ T cell population of AML patients suggest that adoptive transfer of Vδ2 T cells may provide a potential treatment strategy.

### 3.2. Vγ9Vδ2 T Cells Are Cytotoxic Against AML Cell Lines and Primary AML Cells

**Figure 2:**
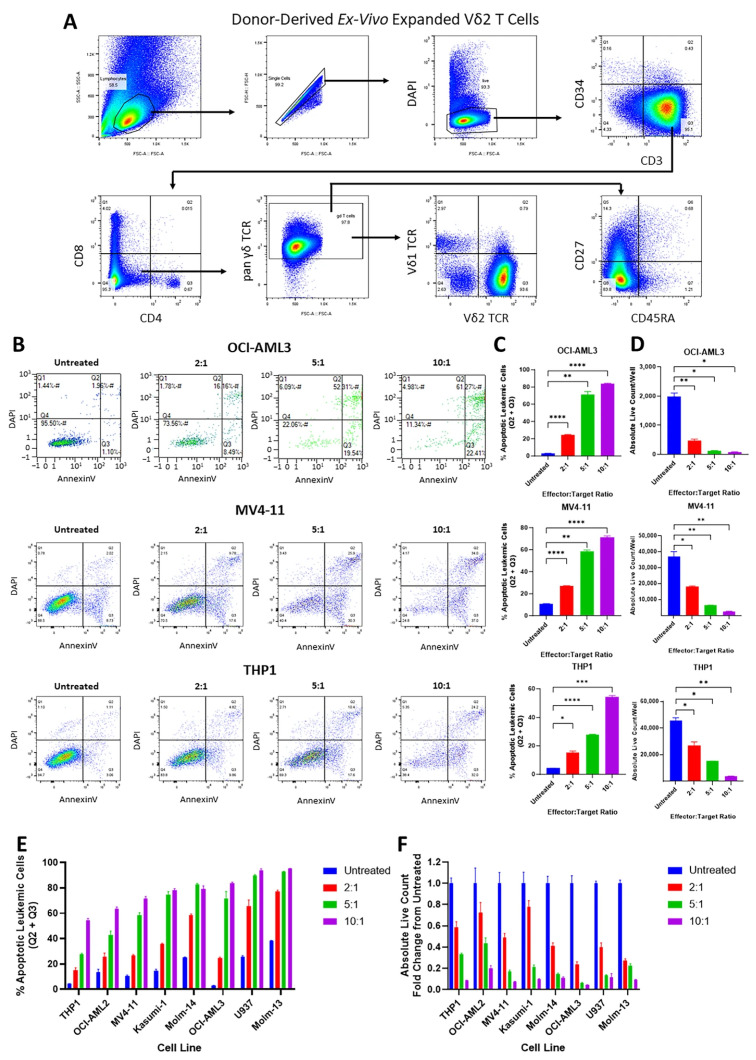
Cytotoxicity of Vγ9Vδ2 T cells against AML cell lines and primary AML cells. Panel (**A**): Representative plots showing the subtype and memory phenotype profile of the donor-derived expanded Vγ9Vδ2 T cells. Panel (**B**): Representative dot plots showing the apoptosis of leukemic cell lines OCI-AML3, MV4-11, and THP1 after 20 h co-culture with Vγ9Vδ2 T cells at the indicated E:T ratios by analyzing the DAPI and Annexin V staining of the CD3-CD33+ population. Panel (**C**): Bar graphs showing the mean percentage of apoptotic leukemic cells by E:T ratio after 20 h co-culture with Vγ9Vδ2 T cells. Panel (**D**): Bar graphs showing the mean absolute live cell count of leukemic cells by E:T ratio after 20 h co-culture with Vγ9Vδ2 T cells. Panel (**E**): Bar graph summarizing the mean percentage of apoptotic leukemic cells for the indicated E:T ratios across AML cell lines. Panel (**F**): Bar graph summarizing the fold-change in the absolute count of live leukemic cells as the E:T ratio increased across AML cell lines. Error bars represent SD. * *p* ≤ 0.05, ** *p* ≤ 0.01, *** *p* ≤ 0.001, **** *p* ≤ 0.0001.

We then sought to investigate the potential of donor-derived Vγ9Vδ2 T cells as an immunotherapy treatment for AML. Flow cytometric analysis confirmed that these cells are primarily Vδ2+ (93.6%) with EM (83.8%) and CM (14.3%) phenotypes (Figure 2A). To understand the cytotoxicity of these Vγ9Vδ2 T cells in vitro, we then performed a flow cytometric apoptosis assay on eight different AML cell lines that had been co-cultured with Vγ9Vδ2 T cells at various E:T ratios for 20 h. After excluding the Vδ2 T cells via their CD3 expression, the leukemic cells were identified through their CD33 expression and analyzed for their apoptosis through DAPI and Annexin V staining. The results show that the percentage of apoptotic leukemic cells increased, while the absolute live leukemic cell count decreased in an E:T ratio-dependent manner (Figure 2B–F).

**Figure 3:**
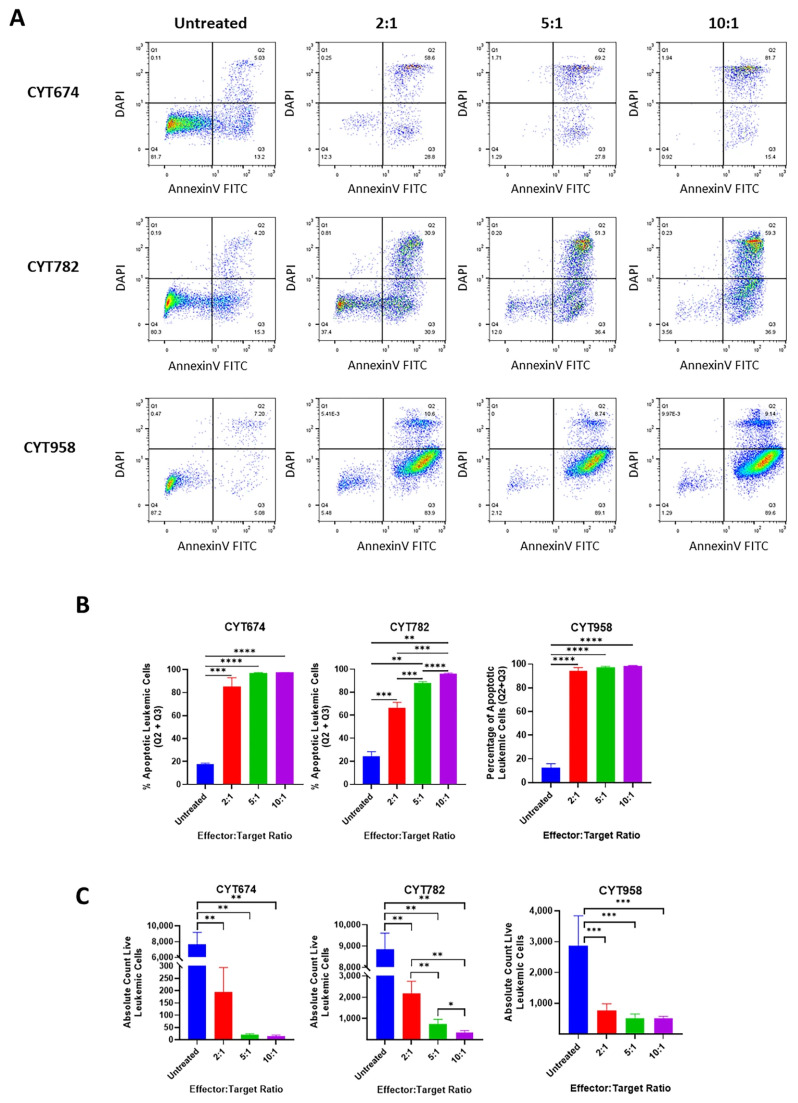
Cytotoxicity of Vγ9Vδ2 T cells against primary AML cells. Primary AML cells from 3 patients (CYT674, CYT782, and CYT958) were co-cultured with Vγ9Vδ2 T cells at the indicated E:T ratios for 20 h. Panel (**A**): Representative dot plots showing the apoptosis of primary AML cells by analyzing the DAPI and Annexin-V staining of the CD3-CD33+ population. Panel (**B**): Bar graphs showing the mean percentage of apoptotic primary AML cells by E:T ratio. Panel (**C**): Bar graphs showing the mean absolute live cell count of primary AML cells after co-culturing at each E:T ratio. Error bars represent SD. * *p* ≤ 0.05, ** *p* ≤ 0.01, *** *p* ≤ 0.001, **** *p* ≤ 0.0001.

The cytotoxicity of these Vγ9Vδ2 T cells was also tested on primary AML cells derived from three AML patients. Again, both the percentage of apoptotic leukemic cells and the absolute live leukemic cell count indicated E:T ratio-dependent killing of primary AML cells (Figure 3). Overall, donor-derived Vγ9Vδ2 T cells effectively induced apoptosis in AML cell lines and primary AML cells, indicating their strong cytotoxic potential.

### 3.3. Vγ9Vδ2 T Cells Alone Extend Survival in Aggressive AML Xenograft Model

**Figure 4:**
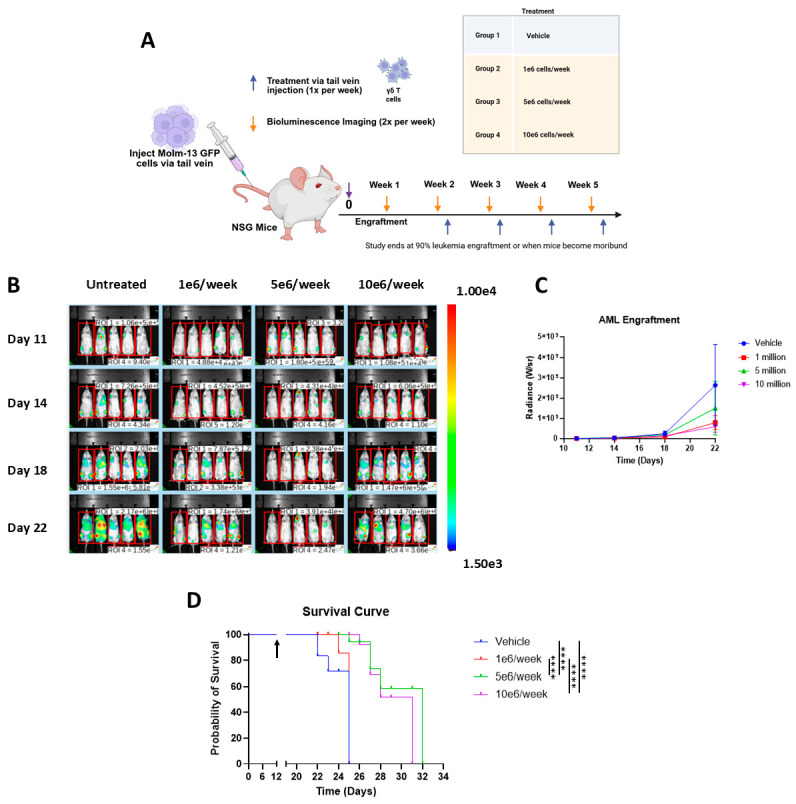
Vγ9Vδ2 T cell therapy prolongs survival in an AML xenograft model. Panel (**A**): Experimental design for dose-escalation study of Vγ9Vδ2 T cell therapy in AML xenograft model established in NSG mice using Molm-13 GFP *Luc* cells. Mice were treated once a week with 1 × 10^6^, 5 × 10^6^, or 1 × 10^7^ Vγ9Vδ2 T cells, or a vehicle (PBS). Panel (**B**): Bioluminescence images showing leukemia engraftment over time measured as radiance. Panel (**C**): Line graph depicting the mean leukemia engraftment over time for each treatment group represented as mean radiance. Panel (**D**): Survival plot comparing the survival curve for each treatment group. The arrow indicates treatment start on day 12. Error bars represent SD. **** *p* ≤ 0.0001.

As demonstrated in Figure 2E,F, Molm-13 cells were sensitive to Vγ9Vδ2 T cell-mediated apoptosis in vitro. To understand the potential of Vγ9Vδ2 T cells for immunotherapy of AML, an AML xenograft model was established in NSG mice using Molm-13 GFP *Luc* cells (Figure 4A) and a dose-escalation study was carried out by treating the mice with various numbers of Vγ9Vδ2 T cells. Molm-13 xenografts were aggressive and fast-growing in NSG mice as monitored through bioluminescence imaging (Figure 4B). Treatment with Vγ9Vδ2 T cells slowed down the Molm-13 growth (Figure 4C); however, there is no statistical difference between groups (Figure 4C). Both treatments with 5 × 10^6^ or 1 × 10^7^ VγVδ2 T cells per week extended the median survival to 27 days from 23 days in the group treated with vehicle (PBS) (*p* < 0.0001) (Figure 4D). While Vγ9Vδ2 T cell treatment did not significantly suppress leukemia burden, it significantly prolonged the survival of the mice, indicating a therapeutic benefit in vivo.

### 3.4. Vγ9Vδ2 T Cells Are Cytotoxic to Venetoclax-Resistant AML Cells and Vγ9Vδ2 T Cells Alone or in Combination with Venetoclax Extends Survival in Venetoclax-Resistant AML Xenograft Model

**Figure 5:**
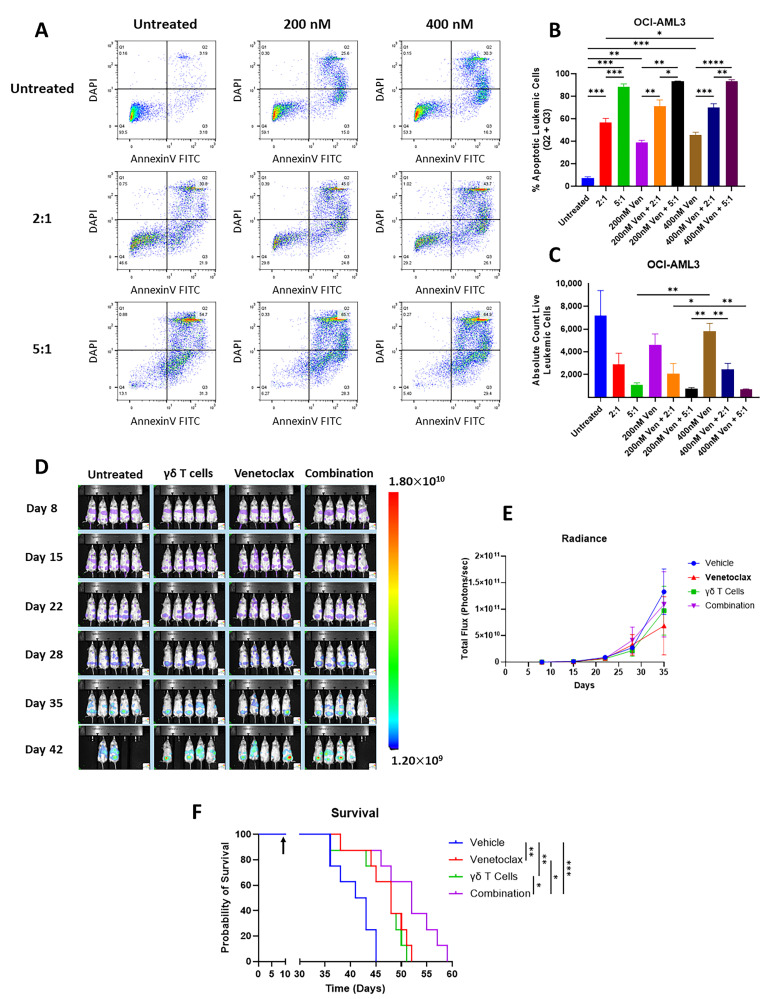
VγVδ2 T cells and venetoclax combination therapy extend survival in a venetoclax-resistant AML xenograft model. Panel (**A**): OCI-AML3 cells were treated for 48 h with 0 nM, 200 nM, or 400 nM venetoclax and Vγ9Vδ2 T cells at E:T ratios of 0:1, 2:1, or 5:1 for 20 h. Apoptosis of OCI-AML3 cells is measured by analyzing the DAPI and Annexin-V staining of the CD3-CD33+ population. Panel (**B**): Bar graph of the mean percentage of apoptotic OCI-AML3 cells for each treatment group. Panel (**C**): Bar graph of the mean absolute count of live OCI-AML3 cells for each treatment group. Panel (**D**): OCI-AML3 GFP *Luc* xenograft-bearing mice were treated once weekly with 5 × 10^6^ Vδ2 T cells and twice weekly with venetoclax for 6 weeks. Bioluminescence images show leukemia engraftment over time in a representative cage of 5 mice for each treatment group. Panel (**E**): Line graph of the mean leukemia engraftment over time for each treatment group, represented as the mean radiance. Panel (**F**): Survival plot showing the survival curve for each treatment group. The arrow indicates the treatment starts on day 9. Error bars represent SD. * *p* ≤ 0.05, ** *p* ≤ 0.01, *** *p* ≤ 0.001, **** *p* ≤ 0.0001.

Venetoclax is commonly used as a universal sensitizer in venetoclax-based combination therapies such as VEN + HMA, which are the current frontline therapies for AML patients who are unable to tolerate intensive chemotherapy regimens. However, venetoclax resistance is not uncommon [40]. It is of great therapeutic interest to find out whether Vγ9Vδ2 T cells are cytotoxic to venetoclax-resistant AML and whether Vγ9Vδ2 T cells may work in the presence of venetoclax. The OCI-AML3 cell line is known to be resistant to venetoclax [41]. Indeed, this was visible in our own results, as the difference in the effect of 200 nM vs. 400 nM venetoclax treatment on OCI-AML3 cells was nonsignificant through both the percentage of apoptotic cells (*p* = 0.1657) and absolute live count (*p* = 0.8084) (Figure 5A–C). In contrast, Vγ9Vδ2 T cells alone efficiently induced apoptosis in OCI-AML3 in an E:T ratio-dependent manner (7.31% ± 1.15 at 0:1, 56.80% ± 3.67 at 2:1, and 88.73 ± 2.42 for 5:1) (Figure 5B), suggesting that the killing mechanism of Vγ9Vδ2 T cells is effective against venetoclax-resistant cells. More interestingly, in the presence of 200 nM and 400 nM venetoclax, Vγ9Vδ2 T cells remained functional and induced significantly more apoptosis than venetoclax alone (Figure 5B), which was further confirmed by the absolute live leukemic cell count (Figure 5C), indicating the potential of integrating Vγ9Vδ2 T cells into a venetoclax-based therapy.

To further investigate the feasibility of a venetoclax + Vγ9Vδ2 T cell combination therapy, we established an AML xenograft model using OCI-AML3 GFP *Luc* cells in NSG mice. The mice were treated twice weekly with 100 mg/kg venetoclax orally and once weekly tail vein injections of 5 × 10^6^ Vγ9Vδ2 T cells. We monitored leukemia engraftment with bioluminescence imaging (Figure 5D). Although there was no significant difference observed in leukemia growth between groups (Figure 5E), there was significant extended survival in all treatment groups, which increased from a median survival of 42 days in the control group to 48 days in the Vγ9Vδ2 T cell therapy group (*p* = 0.0091), 48 days in the venetoclax group (*p* = 0.0061), and 52 days in the combination group (*p* = 0.0009) (Figure 5F). Survival for the combination group was also significantly longer than that of either of the single-agent group (vs. Vγ9Vδ2 T cell therapy: *p* = 0.0257; vs. venetoclax: *p* = 0.0497).

## 4. Discussion

In this study, we have first studied the profile of peripheral blood γδ T cells of AML patients who were unsuitable for intensive chemotherapy and enrolled on the HMA–Ven protocol. Within the T cell population, the CD8+ T cell population was significantly smaller in the AML cohort compared to in healthy donors, which corroborates the known dysfunction and exhaustion of CD8+ T cells in AML patients [42,43]. Similarly, we observed a smaller population of γδ T cells in AML patients. Moreover, the TEMRA phenotype was more prevalent in the γδ T cell population in the AML cohort, while the CM phenotype was less, which corresponds with the results of a previous study on the low expansion capability of γδ T cells in CLL patients [36]. Besides sculpting the memory phenotype, we suspect that AML may cause shifting in the γδ T cell subtypes. In previous studies of melanoma patients, a higher frequency of Vδ1 T cells and a lower frequency of Vδ2 T cells in peripheral blood at baseline have been reported, and the Vδ1 to Vδ2 ratio has been negatively associated with survival [25,26]. Similarly, in this study, we observe the decrease in Vδ2 T cell frequency in AML patients; however, the significantly increased subtype was Vδ1-Vδ2-γδ T cells, but not Vδ1 T cells. The mechanism behind such different γδ T cell subtype shifts in AML patients remains unclear, but it could be due to the expansion of other γδ T cells, such as Vδ3 T cells and Vδ5 T cells [44], or an increase in γδ T regulatory cells (Tregs), such as γδ T17 cells and FoxP3+ γδ Tregs, which have been associated with immunosuppression [21]. Thus, a recurring theme in the change in γδ T cell profile in melanoma and AML patients is the decreased frequency of Vδ2 T cells, suggesting that replenishing the patients with Vδ2 T cells may be a potential treatment.

To investigate the feasibility of using Vδ2 cells, more specifically Vγ9Vδ2 T cells, for immunotherapy of AML, we have further studied the susceptibility of AML cells to Vγ9Vδ2 T cell-mediated cytotoxicity. Since Vγ9Vδ2 T cells recognize cancer cells in an MHC-independent manner, it is possible to develop such treatment using donor-derived Vγ9Vδ2 T cells. Moreover, GMP-grade donor-derived Vγ9Vδ2 T cells are readily available on a clinically relevant scale as an “off-the-shelf” cryopreserved treatment. Our data show that donor-derived Vγ9Vδ2 T cells efficiently induced E:T ratio-dependent apoptosis in all eight AML cell lines and primary AML cells derived from three patients, as evidenced by both the percentage of apoptotic cells and the absolute count of live leukemic cells. Our findings align with a previous study highlighting the efficacy of Vγ9Vδ2 T cells in targeting chemotherapy-resistant AML cells, where Wu et al. found that Vγ9Vδ2 T cells can efficiently kill drug-resistant AML cell lines and primary blasts from refractory AML patients through the CD226-ERK1/2-LAMP1 signaling pathway [45]. Importantly, treatment with Vγ9Vδ2 T cells as a single agent extended the survival of mice engrafted with an aggressive AML cell line.

Despite the improvement of mouse survival, it would be beneficial for future studies to examine how the effect of Vγ9Vδ2 T cells on AML could be improved. Though we observed dramatic effects exerted by the Vγ9Vδ2 T cells on AML in vitro, there was no visible improvement in the leukemia burden in vivo. A recent article by Jiang et al. observed that higher expression of the immune checkpoint receptor TIGIT in γδ T cells, including in the Vδ2 subset, is correlated with poorer outcomes in AML [46]. As such, it may be important to compare the expression of TIGIT and other markers associated with effector cell exhaustion and activation, such as PD-1 and NKG2D, on Vγ9Vδ2 T cells at various time points after exposure to AML and consider potential combinatorial therapeutic options, such as with an anti-TIGIT agent or with cytarabine, an approved chemotherapy known to increase the NKG2D ligand expression on AML [46,47].

For AML patients unfit for intensive chemotherapy, venetoclax-based combination therapies (VEN + HMA) have become a standard of care. However, failure of such frontline regimens in AML patients due to venetoclax resistance is not uncommon, and such patients have dismal overall survival despite salvage therapy. Thus, it is clinically relevant to find out the effect of Vγ9Vδ2 T cells on venetoclax-resistant AML cell lines such as OCI-AML3 [40,41]. Our findings suggested that Vγ9Vδ2 T cells efficiently killed OCI-AML3 in vitro and extended the survival of mice engrafted with OCI-AML3, suggesting the feasibility of using Vγ9Vδ2 T cells as a new salvage therapy for venetoclax-resistant AML patients. To further explore the possibility of incorporating Vγ9Vδ2 T cells in a venetoclax-based combination therapy, we also tested the cytotoxicity of Vγ9Vδ2 T cells in the presence of venetoclax and found that these cells remained cytotoxic to OCI-AML3 cells. Most notably, we observed the venetoclax and Vγ9Vδ2 T cells combination exerted a additive effect on the survival of the OCI-AML3 xenograft mice, indicating a potential clinical benefit of the combination regimen. This corroborates a study presented at the 2024 American Society of Hematology (ASH) meeting reporting that venetoclax treatment significantly enhanced the anti-tumor efficacy of γδ T cells against AML cell lines and patient-derived cells, achieving a 49.21% killing efficiency compared to 5.06% with untreated cells. The authors also reported that this combination decreased tumor burden in THP-1-bearing mice and upregulated activation markers such as CD25 and CD69, as well as cytotoxic molecules like NKG2D and DNAM-1 on drug-treated γδ T cells [48]. Unlike the singular mechanism of action of venetoclax (inhibiting BCL-2), Vγ9Vδ2 T cells have a variety of mechanisms that they may use to target AML cells, including granule-mediated cytotoxicity, TNF-mediated cytotoxicity, and the death receptor pathways [14]. The abundant mechanisms of action of Vγ9Vδ2 T cells may be useful in overcoming venetoclax resistance. For example, the upregulation of MCL-1 has been identified as a mechanism of venetoclax resistance and targeting MCL-1 directly and indirectly is being pursued as salvage therapy options [40]. To this end, granzyme B released by cytotoxic effectors can induce degradation of MCL-1 and trigger apoptosis [49,50]. As such, combining Vγ9Vδ2 T cells with venetoclax and established VEN + HMA regimens offers the potential to overcome venetoclax resistance and may provide a promising therapeutic strategy for venetoclax-resistant AML.

## 5. Conclusions

The aberrant subtype and memory phenotype profiles of peripheral blood γδ T cells in AML patients present an intervention opportunity via the adoptive transfer of Vδ2 T cells. Donor-derived Vγ9Vδ2 T cells can efficiently mediate cytotoxicity in AML cells, including those with venetoclax resistance. Vγ9Vδ2 T cells remain cytotoxic in the presence of venetoclax, and treatment using Vγ9Vδ2 T cells as a single agent or in combination with venetoclax significantly extended the survival in AML xenograft models. Overall, this study demonstrates that Vγ9Vδ2 T cells may provide a promising “off-the-shelf” immunotherapy approach for treating AML patients, particularly those with venetoclax-resistant disease.

## Figures and Tables

**Table 1:** Percentage of T cell subsets out of total CD3+ cells in AML patients and healthy donors.

Population	Healthy Donor		AML		Difference	
	Mean %	SD %	Mean %	SD %	Mean %	*p*-Value
CD8+ T cells	41.32	9.29	27.97	13.74	−13.35	0.0004
CD8+ CD4+ T cells	3.13	4.85	1.13	1.25	−2.00	ns
CD4+ T cells	50.55	10.88	56.06	20.63	5.51	ns
CD4- CD8- T cells	5.00	2.90	14.84	19.37	9.84	0.031
γδ T cells	57.03	19.60	39.62	21.14	−17.41	0.0039
Vδ1 T cells	20.70	19.50	28.63	29.67	7.93	ns
Vδ2 T cells	66.21	22.54	30.59	27.46	−35.62	0.0023
Vδ1- Vδ2- T cells	12.37	9.04	37.09	32.96	24.72	0.006
Naïve γδ T cells	18.54	9.42	22.28	15.22	3.74	ns
CM γδ T cells	48.45	14.36	18.95	16.78	−29.50	<0.0001
EM γδ T cells	12.40	8.49	20.83	22.43	8.43	ns
TEMRA γδ T cells	20.63	16.10	37.94	24.94	17.31	0.0163

Abbreviations: AML, acute myeloid leukemia; CM, central memory; EM, effector memory; ns, not significant; TEMRA, terminally differentiated effector memory; SD, standard deviation.

## Data Availability

The data that support the findings of this study are available from the corresponding author upon reasonable request.

## Supplementary Materials

The following supporting information can be downloaded at https://www.mdpi.com/article/10.3390/cancers17193166/s1, Supplementary Table S1. Patient Characteristics. Supplementary Table S2. Viability of AML Patient-derived PBMCs. Supplementary Figure S1. Peripheral blood T cell subsets based on CD4 and CD8 expression in AML patients and healthy donors. Scatter plot showing the various T cell subsets through mean and SD. * *p* ≤ 0.05, ** *p* ≤ 0.01, *** *p* ≤ 0.001, **** *p* ≤ 0.0001.

## References

[1] Vakiti A., Reynolds S.B., Mewawalla P. (2024). Acute Myeloid Leukemia.

[2] Short N.J., Rytting M.E., Cortes J.E. (2018). Acute myeloid leukaemia. Lancet.

[3] Kolitz J.E., Larson R.A., Rosmarin A.G. (2022). Acute Myeloid Leukemia in Adults: Overveiw.

[4] Ohmoto A., Fuji S. (2023). Clinical status of induction therapy incorporating a hypomethylating agent for newly diagnosed adult acute myeloid leukemia compared to the standard 7 + 3 regimen. Expert Rev. Hematol..

[5] Heuser M., Fernandez C., Hauch O., Klibanov O.M., Chaudhary T., Rives V. (2023). Therapies for acute myeloid leukemia in patients ineligible for standard induction chemotherapy: A systematic review. Future Oncology.

[6] Medeiros B.C., Satram-Hoang S., Hurst D., Hoang K.Q., Momin F., Reyes C. (2015). Big data analysis of treatment patterns and outcomes among elderly acute myeloid leukemia patients in the United States. Ann. Hematol..

[7] Döhner H., Wei A.H., Appelbaum F.R., Craddock C., DiNardo C.D., Dombret H., Ebert B.L., Fenaux P., Godley L.A., Hasserjian R.P. (2022). Diagnosis and management of AML in adults: 2022 recommendations from an international expert panel on behalf of the ELN. Blood.

[8] DiNardo C.D., Jonas B.A., Pullarkat V., Thirman M.J., Garcia J.S., Wei A.H., Konopleva M., Döhner H., Letai A., Fenaux P. (2020). Azacitidine and Venetoclax in Previously Untreated Acute Myeloid Leukemia. N. Engl. J. Med..

[9] Maiti A., Rausch C.R., Cortes J.E., Pemmaraju N., Daver N.G., Ravandi F., Garcia-Manero G., Borthakur G., Naqvi K., Ohanian M. (2021). Outcomes of relapsed or refractory acute myeloid leukemia after frontline hypomethylating agent and venetoclax regimens. Haematologica.

[10] Xu Z., Huang X. (2021). Cellular immunotherapy for hematological malignancy: Recent progress and future perspectives. Cancer Biol. Med..

[11] Sterner R.C., Sterner R.M. (2021). CAR-T cell therapy: Current limitations and potential strategies. Blood Cancer J..

[12] Goldenson B.H., Kaufman D.S. (2021). Into the multiverse of gene edited NK cell-based therapeutic strategies. Cell Stem Cell.

[13] Paul S., Lal G. (2016). Regulatory and effector functions of gamma-delta (gammadelta) T cells and their therapeutic potential in adoptive cellular therapy for cancer. Int. J. Cancer.

[14] Ou L., Wang H., Liu Q., Zhang J., Lu H., Luo L., Shi C., Lin S., Dong L., Guo Y. (2021). Dichotomous and stable gamma delta T-cell number and function in healthy individuals. J. Immunother. Cancer.

[15] Deseke M., Prinz I. (2020). Ligand recognition by the γδ TCR and discrimination between homeostasis and stress conditions. Cell. Mol. Immunol..

[16] Gentles A.J., Newman A.M., Liu C.L., Bratman S.V., Feng W., Kim D., Nair V.S., Xu Y., Khuong A., Hoang C.D. (2015). The prognostic landscape of genes and infiltrating immune cells across human cancers. Nat. Med..

[17] Klyuchnikov E., Badbaran A., Massoud R., Fritsche-Friedland U., Janson D., Ayuk F., Wolschke C., Bacher U., Kröger N. (2021). Enhanced Immune Reconstitution of γδ T Cells after Allogeneic Stem Cell Transplantation Overcomes the Negative Impact of Pretransplantation Minimal Residual Disease-Positive Status in Patients with Acute Myelogenous Leukemia. Transplant. Cell. Ther..

[18] Godder K.T., Henslee-Downey P.J., Mehta J., Park B.S., Chiang K.-Y., Abhyankar S., Lamb L.S. (2007). Long term disease-free survival in acute leukemia patients recovering with increased γδ T cells after partially mismatched related donor bone marrow transplantation. Bone Marrow Transplant..

[19] Minculescu L., Marquart H.V., Ryder L.P., Andersen N.S., Schjoedt I., Friis L.S., Kornblit B.T., Petersen S.L., Haastrup E., Fischer-Nielsen A. (2019). Improved Overall Survival, Relapse-Free-Survival, and Less Graft-vs.-Host-Disease in Patients with High Immune Reconstitution of TCR Gamma Delta Cells 2 Months After Allogeneic Stem Cell Transplantation. Front. Immunol..

[20] Perko R., Kang G., Sunkara A., Leung W., Thomas P.G., Dallas M.H. (2015). Gamma Delta T Cell Reconstitution is Associated with Fewer Infections and Improved Event-Free Survival after Hematopoietic Stem Cell Transplantation for Pediatric Leukemia. Biol. Blood Marrow Transplant..

[21] Zhao Y., Niu C., Cui J. (2018). Gamma-delta (gammadelta) T cells: Friend or foe in cancer development?. J. Transl. Med..

[22] Almeida A.R., Correia D.V., Fernandes-Platzgummer A., da Silva C.L., da Silva M.G., Anjos D.R., Silva-Santos B. (2016). Delta One T Cells for Immunotherapy of Chronic Lymphocytic Leukemia: Clinical-Grade Expansion/Differentiation and Preclinical Proof of Concept. Clin. Cancer Res..

[23] Siegers G.M., Dhamko H., Wang X.-H., Mathieson A.M., Kosaka Y., Felizardo T.C., Medin J.A., Tohda S., Schueler J., Fisch P. (2011). Human Vδ1 γδ T cells expanded from peripheral blood exhibit specific cytotoxicity against B-cell chronic lymphocytic leukemia-derived cells. Cytotherapy.

[24] Kimura Y., Nagai N., Tsunekawa N., Sato-Matsushita M., Yoshimoto T., Cua D.J., Iwakura Y., Yagita H., Okada F., Tahara H. (2016). IL-17A-producing CD30+ Vδ1 T cells drive inflammation-induced cancer progression. Cancer Sci..

[25] Wistuba-Hamprecht K., Martens A., Haehnel K., Foppen M.G., Yuan J., Postow M.A., Wong P., Romano E., Khammari A., Dreno B. (2016). Proportions of blood-borne Vδ1+ and Vδ2+ T-cells are associated with overall survival of melanoma patients treated with ipilimumab. Eur. J. Cancer.

[26] Wistuba-Hamprecht K., Di Benedetto S., Schilling B., Sucker A., Schadendorf D., Garbe C., Weide B., Pawelec G. (2016). Phenotypic characterization and prognostic impact of circulating γδ and αβ T-cells in metastatic malignant melanoma. Int. J. Cancer.

[27] Rong L., Li K., Li R., Liu H.M., Sun R., Liu X.Y. (2016). Analysis of tumor-infiltrating gamma delta T cells in rectal cancer. World J. Gastroenterol..

[28] Lee D., Dunn Z.S., Guo W., Rosenthal C.J., Penn N.E., Yu Y., Zhou K., Li Z., Ma F., Li M. (2023). Unlocking the potential of allogeneic Vδ2 T cells for ovarian cancer therapy through CD16 biomarker selection and CAR/IL-15 engineering. Nat. Commun..

[29] Karunakaran M.M., Willcox C.R., Salim M., Paletta D., Fichtner A.S., Noll A., Starick L., Nöhren A., Begley C.R., Berwick K.A. (2020). Butyrophilin-2A1 Directly Binds Germline-Encoded Regions of the Vγ9Vδ2 TCR and Is Essential for Phosphoantigen Sensing. Immunity.

[30] Herrmann T., Fichtner A.S., Karunakaran M.M. (2020). An Update on the Molecular Basis of Phosphoantigen Recognition by Vγ9Vδ2 T Cells. Cells.

[31] Rao A., Agrawal A., Borthakur G., Battula V.L., Maiti A. (2024). Gamma delta T cells in acute myeloid leukemia: Biology and emerging therapeutic strategies. J. Immunother. Cancer.

[32] Vydra J., Cosimo E., Lesný P., Wanless R.S., Anderson J., Clark A.G., Scott A., Nicholson E.K., Leek M. (2023). A Phase I Trial of Allogeneic γδ T Lymphocytes From Haploidentical Donors in Patients with Refractory or Relapsed Acute Myeloid Leukemia. Clin. Lymphoma Myeloma Leuk..

[33] Anane L.H., Edwards K.M., Burns V.E., van Zanten J.J.V., Drayson M.T., Bosch J.A. (2010). Phenotypic characterization of γδ T cells mobilized in response to acute psychological stress. Brain Behav. Immun..

[34] Caccamo N., Meraviglia S., Ferlazzo V., Angelini D., Borsellino G., Poccia F., Battistini L., Dieli F., Salerno A. (2005). Differential requirements for antigen or homeostatic cytokines for proliferation and differentiation of human Vγ9Vδ2 naive, memory and effector T cell subsets. Eur. J. Immunol..

[35] Angelini D.F., Borsellino G., Poupot M., Diamantini A., Poupot R., Bernardi G., Poccia F., Fournié J.J., Battistini L. (2004). FcγRIII discriminates between 2 subsets of Vγ9Vδ2 effector cells with different responses and activation pathways. Blood.

[36] Coscia M., Vitale C., Peola S., Foglietta M., Rigoni M., Griggio V., Castella B., Angelini D., Chiaretti S., Riganti C. (2012). Dysfunctional Vγ9Vδ2 T cells are negative prognosticators and markers of dysregulated mevalonate pathway activity in chronic lymphocytic leukemia cells. Blood.

[37] Maiti A., Peterlin P., Morillo D., Torregrosa-Diaz J.-M., Ulrickson M., Penedo A., De Gassart A., Wieduwild E., Valentin E., Mairesse M. (2025). Abstract CT024: γ9δ2 T-cell (γδTC) activation and azacitidine-venetoclax (AV) for older/unfit adults with newly diagnosed (ND) acute myeloid leukemia (AML) induces high rates of complete remission (CR): Preliminary efficacy, safety, pharmacodynamics (PD) and dose selection of ICT01 in the phase 1 study EVICTION. Cancer Res..

[38] Du S.H., Li Z., Chen C., Tan W.K., Chi Z., Kwang T.W., Xu X.H., Wang S. (2016). Co-Expansion of Cytokine-Induced Killer Cells and Vγ9Vδ2 T Cells for CAR T-Cell Therapy. PLoS ONE.

[39] Vera J., Savoldo B., Vigouroux S., Biagi E., Pule M., Rossig C., Wu J., Heslop H.E., Rooney C.M., Brenner M.K. (2006). T lymphocytes redirected against the kappa light chain of human immunoglobulin efficiently kill mature B lymphocyte-derived malignant cells. Blood.

[40] Ong F., Kim K., Konopleva M.Y. (2022). Venetoclax resistance: Mechanistic insights and future strategies. Cancer Drug Resist..

[41] Reis-Silva C.S.M., Branco P.C., Lima K., Silva F.L., Moreno P.R.H., Guallar V., Costa-Lotufo L.V., Machado-Neto J.A. (2021). Embelin potentiates venetoclax-induced apoptosis in acute myeloid leukemia cells. Toxicol. Vitro.

[42] Knaus H.A., Berglund S., Hackl H., Blackford A.L., Zeidner J.F., Montiel-Esparza R., Mukhopadhyay R., Vanura K., Blazar B.R., Karp J.E. (2018). Signatures of CD8+ T cell dysfunction in AML patients and their reversibility with response to chemotherapy. JCI Insight.

[43] Zhou H., Jia B., Annageldiyev C., Minagawa K., Zhao C., Mineishi S., Ehmann W.C., Naik S.G., Cioccio J., Wirk B. (2023). CD26^low^PD-1^+^ CD8 T cells are terminally exhausted and associated with leukemia progression in acute myeloid leukemia. Front. Immunol..

[44] Barros M.S., de Araújo N.D., Magalhães-Gama F., Pereira Ribeiro T.L., Alves Hanna F.S., Tarragô A.M., Malheiro A., Costa A.G. (2021). γδ T Cells for Leukemia Immunotherapy: New and Expanding Trends. Front. Immunol..

[45] Wu K., Wang L.M., Liu M., Xiu Y., Hu Y., Fu S., Huang H., Xu B., Xiao H. (2021). The CD226-ERK1/2-LAMP1 pathway is an important mechanism for Vγ9Vδ2 T cell cytotoxicity against chemotherapy-resistant acute myeloid leukemia blasts and leukemia stem cells. Cancer Sci..

[46] Jiang S., Zheng S., Yao C., Ning D., Zou S., Zhan J., Lan T., Yi T., Jin Z., Wu X. (2025). Heterogeneity of γδ T-cell subsets and their clinical correlation in patients with AML. Front. Immunol..

[47] Wu Z., Zhang H., Wu M., Peng G., He Y., Wan N., Zeng Y. (2021). Targeting the NKG2D/NKG2D-L axis in acute myeloid leukemia. Biomed. Pharmacother..

[48] Xingchi C., Sun G., Sui Y., Ma W., Zhang Y., Zhu X. (2024). Venetoclax Enhances Human Γδt Cells Anti-Leukemia Immunity through Metabolic Reprogramming. Blood.

[49] Han J., Goldstein L.A., Gastman B.R., Froelich C.J., Yin X.M., Rabinowich H. (2004). Degradation of Mcl-1 by Granzyme B: Implications for bim-mediated mitochondrial apoptotic events. J. Biol. Chemistry.

[50] Catalán E., Jaime-Sánchez P., Aguiló N., Simon M.M., Froelich C.J., Pardo J. (2015). Mouse cytotoxic T cell-derived granzyme B activates the mitochondrial cell death pathway in a Bim-dependent fashion. J. Biol. Chem..

