# Supplementary material for: Donor-Derived Vγ9Vδ2 T Cells for Acute Myeloid Leukemia: A Promising “Off-the-Shelf” Immunotherapy Approach
Source: Cancers (Basel). 2025 Sep 29;17(19):3166. doi: 10.3390/cancers17193166 (PMC12524236; doi:10.3390/cancers17193166)
Supplement: Supplementary file 1 [file cancers-17-03166-s001.zip › cancers-3741955-supplementary.pdf]

Supplementary Table S1. Patient Characteristics.

The letters of the sample code differentiate frozen samples from the experiments for flow cytometric profiling of γδ T cells (UTH) from the fresh samples used for apoptosis assays (CYT). Abbreviations: male (M), female (F), white blood cell count (WBC), overall survival (OS), not available (NA).

Sample Code	Gender	Race	Age at Collection	WBC (K/µL)	Blast %	OS (Months)
UTH1858	M	White	85	2.9	6	2
UTH1900	M	White	92	1.9	NA	8
UTH1135	M	White	76	2.7	NA	78
UTH1520	F	White	80	3.7	34	3
UTH1171	M	White	71	1.5	2	40
UTH1569	M	Other	84	0.9	6	21
UTH1893	M	White	75	1.7	6	33
UTH1488	M	White	75	6.6	9	11
UTH3675	M	White	81	0.7	1	11
UTH3492	F	White	77	2.1	12	46
UTH3176	F	NA	54	1.2	NA	19
UTH3440	F	White	77	0.8	5	44
UTH5682	M	White	75	13.8	15	9
UTH5571	M	Other	68	2.2	21	5
TUH5241	F	White	73	37.9	29	0
UTH5464	M	White	68	1.6	NA	5
UTH5959	M	White	77	5.4	34	42
UTH5193	F	White	77	3.2	34	2
UTH5701	M	White	56	0.5	3	64
UTH5482	F	White	72	1.4	NA	71
UTH5021	M	White	57	9.3	85	2
UTH5571	M	White	80	3.6	3	0
UTH5171	M	White	82	3.3	30	3
UTH5624	M	Black	82	10.4	19	20
UTH5225	F	White	75	1.2	4	0
UTH5451	M	White	74	66.8	94	33
UTH5684	F	White	70	2.1	28	27
UTH5245	F	White	76	10.1	NA	15
UTH5790	M	White	92	3.2	14	12
UTH5624	M	White	82	6.7	14	11
UTH5861	F	White	80	4.7	26	5
UTH5051	M	Black	79	2.2	1	2
UTH5904	F	White	83	1.6	NA	2

UTH5942	M	White	72	5.3	21	5
UTH5591	M	White	81	39.0	15	7
CYT674	F	White	33	11.4	75	NA
CYT782	M	White	62	22.3	86	NA
CYT958	M	White	77	24.3	98	NA

Supplementary Table S2. Viability of AML Patient-derived PBMCs

Sample Code	% Live Cells	Sample Code	% Live Cells	Sample Code	% Live Cells
UTH1858	95.90%	UTH3440	99.20%	UTH5171	89.40%
UTH1900	96.40%	UTH5682	96.10%	UTH5624	96.80%
UTH1135	75.30%	UTH5571	81.40%	UTH5225	67.40%
UTH1520	99.20%	UTH5241	94.70%	UTH5451	92.10%
UTH1171	74.70%	UTH5464	96.70%	UTH5684	94.50%
UTH1569	85.90%	UTH5959	86.30%	UTH5245	93.20%
UTH1893	62.30%	UTH5193	90.40%	UTH5790	87.60%
UTH1488	98.00%	UTH5701	98.90%	UTH5624	74.50%
UTH3675	73.70%	UTH5482	98.20%	UTH5861	65.00%
UTH3492	98.60%	UTH5021	76.30%	UTH5051	85.20%
UTH3176	97.20%	UTH5571	94.60%	UTH5904	70.10%

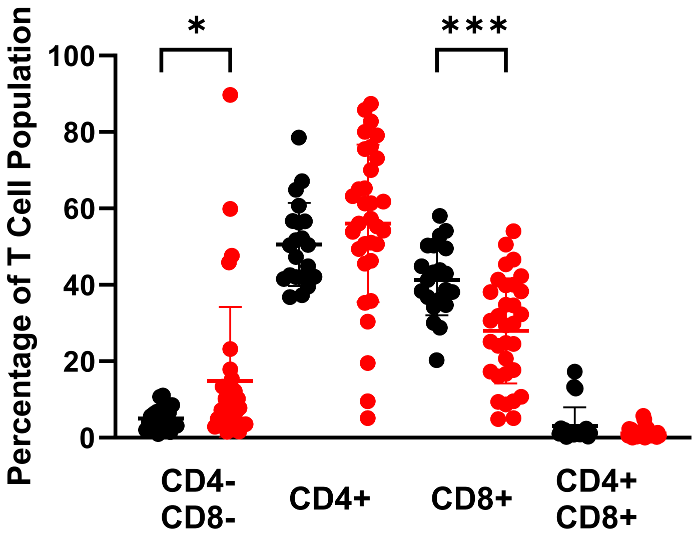

Supplementary Figure S1. Peripheral blood T cell subsets based on CD4 and CD8 expression in AML patients and healthy donors. Scatter plot showing the various T cell subsets through mean and SD. *$p \leq 0.05$, **$p \leq 0.01$, ***$p \leq 0.001$, ****$p \leq 0.0001$.
